# Virtual Medical Modality Implementation Strategies for Patient-Aligned Care Teams to Promote Veteran-Centered Care: Protocol for a Mixed-Methods Study

**DOI:** 10.2196/11262

**Published:** 2018-08-15

**Authors:** Jolie Haun, Margeaux Chavez, Wendy Hathaway, Nicole Antinori, Christine Melillo, Bridget A Cotner, Julie McMahon-Grenz, Brian Zilka, Shilpa Patel-Teague, William Messina, Kim Nazi

**Affiliations:** ^1^ Health Services Research and Development Service Center of Innovation on Disability and Rehabilitation Research James A Haley VA Medical Center Tampa, FL United States; ^2^ Department of Community & Family Health College of Public Health University of South Florida Tampa, FL United States; ^3^ Department of Anthropology University of South Florida Tampa, FL United States; ^4^ Primary Care James A Haley VA Medical Center Tampa, FL United States; ^5^ Veterans Integrated Service Network 8 Network Office St Petersburg, FL United States; ^6^ Veterans and Consumers Health Informatics Office Office of Connected Care Veterans Health Administration, US Department of Veterans Affairs Washington, DC United States

**Keywords:** ambulatory care, health information technology, implementation, medical informatics, veteran, virtual medical modality

## Abstract

**Background:**

The Veterans Health Administration (VHA) is making system-wide efforts to increase integrated use of health information technology (HIT), including My Health*e*Vet (MHV), the Veterans Affairs (VA) electronic patient portal, Vet Link kiosks, telehealth, and mobile apps. Integrated use of HIT can increase individual and system efficiency, maximize resources, and enhance patient outcomes. Prior research indicates that provider endorsement and reinforcement are key determinants of patient adoption of HIT. HIT implementation strategies need to reflect providers’ perspectives to promote adoption and endorsement of these tools; however, providers often lack awareness or are unmotivated to incorporate HIT into clinical care with their patients. When these modalities are used by patients, the approach is often fragmented rather than integrated within and across care settings. Research is needed to identify effective implementation strategies for increasing patient-aligned care team (PACT) member (ie, the VHA’s Patient Centered Medical Home) awareness and motivation to use HIT in a proactive and integrated approach with patients.

**Objective:**

This paper describes the rationale, design, and methods of the PACT protocol to promote proactive integrated use of HIT.

**Methods:**

In Aim 1, focus groups (n=21) were conducted with PACT members (n=65) along with questionnaires and follow-up individual interviews (n=16). In Aim 2, the team collaborated with VA clinicians, electronic health researchers and operational partners to conduct individual expert interviews (n=13), and an environmental scan to collect current and emerging provider-focused implementation tools and resources. Based on Aim 1 findings, a gap analysis was conducted to determine what implementation strategies and content needed to be adapted or developed. Following the adaptation or development of resources, a PACT expert panel was convened to evaluate the resultant content. In Aim 3, a local implementation of PACT-focused strategies to promote integrated use of HIT was evaluated using pre- and postquestionnaire surveys, brief structured interviews, and secondary data analysis with PACT members (n=63).

**Results:**

Study enrollment for Aim 1 has been completed. Aims 1 and 2 data collection and analysis are underway. Aim 3 activities are scheduled for year 3.

**Conclusions:**

This work highlights the practical, technological, and participatory factors involved in facilitating implementation research designed to engage PACT clinical members in the proactive integrated use of HIT. These efforts are designed to support the integrated and proactive use of VA HIT to support clinical care coordination in ways that are directly aligned with PACT member preferences. This study evaluated integrated VA HIT use employing mixed-methods and multiple data sources. Deliverables included PACT-focused strategies to support integrated use of HIT in the ambulatory care setting that will also inform strategy development in other systems of care and support subsequent implementation efforts at regional and national levels.

**Registered Report Identifier:**

RR1-10.2196/11262

## Introduction

### Background

The Department of Veterans Affairs (VA) MyVA Initiative articulates a vision for personalized, proactive, and patient-driven care leveraging information technologies, analytics, and new models of health care delivery [1,2]. To operationalize this vision, the Veterans Health Administration (VHA) is making system-wide efforts to increase the integrated use of health information technology (HIT), including My Health*e*Vet (MHV), VetLink kiosks, telehealth, and mobile apps. Integrated use of HIT can increase individual and system efficiency, maximize resources, and enhance patient outcomes [3]. Prior research indicates that provider endorsement and reinforcement are key determinants of patient adoption of HIT [4,5]. HIT implementation strategies need to reflect providers’ perspectives to promote adoption and endorsement of these tools [6]. Providers often lack awareness or are unmotivated to incorporate HIT into clinical care with their patients [3]. Current use of these modalities is often fragmented rather than integrated within and across care settings [3]. Therefore, research is needed to identify effective implementation strategies for increasing the proactive and integrated use of HIT among patient-aligned care teams (PACTs; VHA’s primary care Patient-Centered Medical Home model).

### Research Aims

This research was responsive to the vision of the VA’s MyVA Initiative and strategic plan for veterans to receive timely and integrated care through enhanced use of HIT. Our short-term goal was to locally develop, deliver, and evaluate implementation strategies with both core PACT members (ie, clerical associates, clinical associates, nurses, providers) and extended PACT members (ie, mental health, nutrition, pharmacy, social work) to increase use of HIT. This research sets the foundation for subsequent implementation efforts at regional and national levels. Long term, this research has potential to inform sustained HIT use by PACTs in a nationwide integrated health care system. We used a community-based participatory research approach [7] and theoretical constructs from the Consolidated Framework for Implementation Research [8] including elements from the Diffusion of Innovations theory [9,10] and the Promoting Action on Research Implementation in Health Services (PARiHS) framework [11] to inform this 3-year concurrent mixed-method [12] implementation study. The research aims were to (1) identify characteristics of PACT members that impact HIT use among high- and low-volume users, (2) develop implementation strategies to promote PACT adoption of HIT, and (3) evaluate local implementation of PACT-focused strategies to promote HIT adoption.

The first aim addressed three research questions: (1) What are the characteristics of high- and low-volume clinical team users? (2) What factors influence use (ie, compatibility, observability, complexity, relative advantage)? and (3) What are PACT member experiences and preferences for using HIT (ie, evidence, context, facilitation)?

The second aim addressed two research questions: (1) What implementation strategies currently exist to support HIT adoption? and (2) What implementation strategies promote HIT adoption?

Finally, the third research aim addressed three research questions: (1) Do implementation strategies reflect PACT member needs and preferences? (2) How can the implementation strategies be refined and improved to support adoption of HIT? and (3) Does exposure to implementation strategies increase PACT use of HIT? The third research aim also addressed two hypotheses: (1) Exposure to implementation strategies will increase PACT members’ self-reported value, intention, and use of HIT, and (2) Exposure to implementation strategies will significantly increase PACT use of HIT.

Intended outcomes of this study were to develop novel PACT-focused strategies to advance implementation science, implement a pioneering protocol to objectively evaluate integrated HIT use employing secondary data sources, and support operational efforts in the expansion of HIT within VHA.

### Background on the VA’s Health Information Technology

The VA’s HIT are central components for delivering personalized, proactive, and patient-driven care. My Health*e*Vet, mobile apps, VetLink kiosks, and telehealth are core virtual resource technologies designed to increase patient access, participation in care, and self-care management. The VA is vested in supporting veteran and provider use of virtual resources to improve patient outcomes [13,14] and promote efficient system utilization. In alignment with the strategic plan for Digital Services put forth by the VA Secretary’s Office, HIT provides an integrated approach to delivering virtual care throughout the VHA system of care. This integrated HIT system is an important interface to support increased veteran access to the VHA, Veterans Benefits Administration, and National Cemetery Administration.

Patients value virtual health care delivery [15-19]. More than 4 million veterans are registered MHV users (Veterans and Consumers Health Informatics Office, US VA, unpublished data 2017), and this number continues to increase. VA’s multiple HIT platforms (eg, mobile, Web, kiosks) are likely to become the primary interface for patients and providers. Virtual communication between patients and providers facilitate information sharing, patient-centered communication, and coordination of care [11,20]. Use of HIT extends the focus of care beyond the acute care setting to support the ongoing health maintenance, medical treatment, prevention of secondary complications and comorbidities, and psychosocial and community reintegration support of veterans [12,21]. Despite the benefits of HIT, limited information exists about team user preferences for integrating multiple HIT in their clinical practice. The benefits of proactive integrated use have important unexplored potential.

Although veterans drive HIT use based on their preferences and needs, VHA provider engagement is critical for the promotion of sustained and integrated use [4,5,22-24]. Providers can increase HIT use by encouraging patients to enroll and use these resources [3,15,16,25]. Providers may impede use by actively discouraging or passively failing to address patient needs [16,17,26]. The value of exploring the needs and preferences of PACT members was to effectively promote their integrated use of HIT to meet the health care delivery needs of veterans.

### Patient-Aligned Care Team Care Delivery Model and Virtual Medical Modality Use

The VHA PACT initiative was implemented between 2010-2014 to achieve team-based care, improve access, and provide comprehensive care management for more than 5 million veterans for primary care needs [19]. The PACT model emphasizes care delivery by a “team” typically comprising a physician, nurse, clinical associate, and clerical associate. Extended PACT members represent specialty service areas. Since implementation of PACT, the use of HIT has increased. Even with increased use, HIT is still not used to its full potential as a tool to improve veteran access to care. From 2009 (pre-PACT) to September 2012, the following trends have been observed: (1) phone encounter rates increased more than 10-fold for patients assigned to a primary care provider (*P*≤.01 each) [27], (2) the number of patients using telehealth increased from 38,747 (0.8% patients) to 70,486 (1.4% patients; *P*≤.01), and (3) the number of authenticated VA patients with enhanced access to all MHV features increased from 3% of 4,759,668 primary care patients to 13% of 5,163,531 primary care patients (*P*≤.01). Primary care patients using secure messaging (SM) increased from 0.07 per 1000 in 2009 to 22.8 per 1000 in 2012 (*P*≤.01) [19]. It is critical that use of these resources is maximized to realize their potential in delivering patient-driven care.

This study focused on PACT members to understand the personal, contextual, and other factors that impact use of HIT. Based on clinical partner feedback, we originally focused on data collection from PACT because they are the driving force of HIT use among extended PACT members (eg, SM). However, snowball sampling was used to identify noncore PACT members who drive HIT use, to create a holistic dataset from all PACT members.

### Adoption of Health Information Technology as an Integrated System

This study leveraged previous efforts by VHA researchers to support the implementation of individual HIT (eg, Blue Button, SM). As part of this research, we synthesized individual evidence-based strategies to create a more holistic approach to support the use of HIT as an integrated system. We conducted a preliminary review to develop and refine our proposed aims informed by our previous work in this area. While much of the work in this area is recent and not yet published, our review clearly identified the need for additional research to better understand providers’ perspectives in implementing HIT and created a starting point for leveraging existing materials to develop PACT-focused implementation strategies.

Implementation of HIT often focuses on a single HIT [18,28-31]. Few strategies have focused on supporting implementation of multiple HIT platforms, and few are specific to the needs of PACT, both of which represent a limitation in these approaches. If strategies are focused on single HIT, siloed use of HIT is perpetuated. Furthermore, if strategies are not specific to PACT, there is a gap between HIT capacity and specific use for PACT in clinical settings. To maximize impact, HIT needs to be used as an integrated system in ways that are most appropriate for PACT, for example, a clinical team can receive a secure message (ie, email) from a patient reporting an increase in blood pressure. The team’s licensed practical nurse reviews the message and determines that the patient needs additional monitoring. She or he then alerts the team’s registered nurse (RN) who uses telehealth technology to remotely collect the patient’s vitals over the next several days. The patient’s blood pressure remains high so the RN schedules a remote appointment using televideo technology to follow up. During this appointment the patient asks for blood pressure medication to be refilled and anxiety medication renewed. The RN alerts the physician to authorize the prescription refill and alerts the team’s clerk to schedule an in-person appointment to address the renewal. At the appointment, the physician prescribes use of a relevant mobile app to help the patient manage their health conditions. The provider can then document the education in the Veteran’s electronic health record for other providers to access and then renew the patient’s prescription using the electronic system. This example is only one example of proactive integrated use; it is critical to develop and disseminate a comprehensive set of these best practices. Though generic HIT implementation strategies exist for delivering education to clinical care team members to increase awareness and support sustained use, they are not comprehensive, do not promote integrated use, and are not PACT specific.

## Methods

### Design and Overview

**Figure 1:**
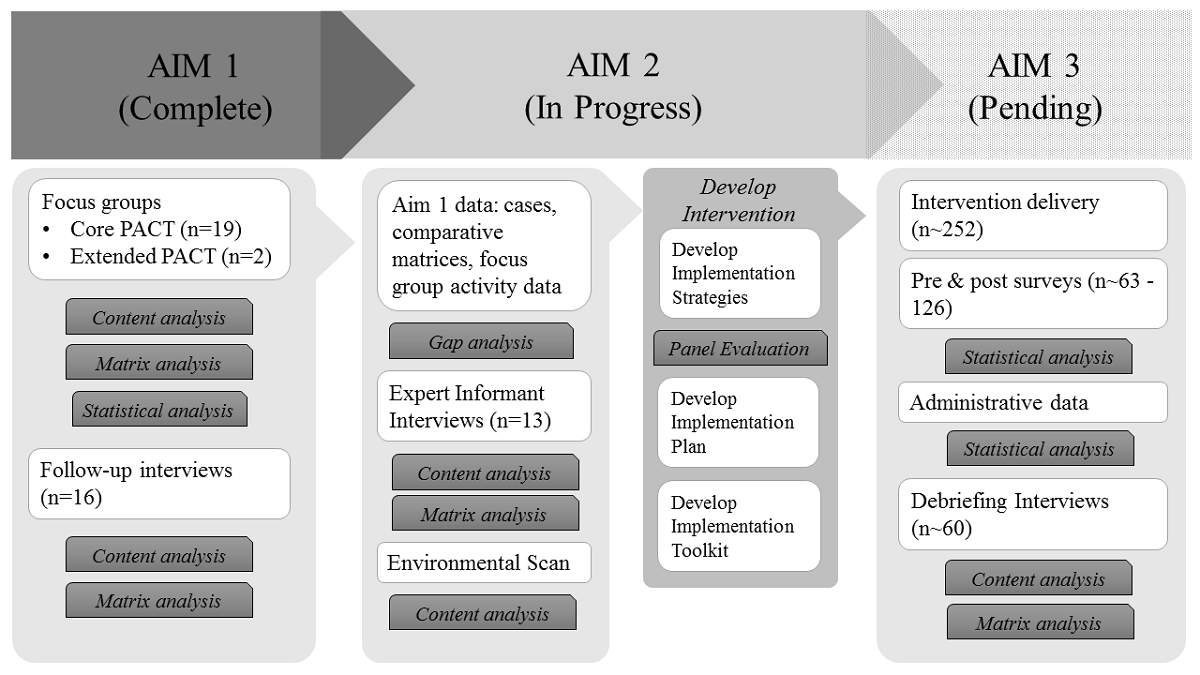
Study flow chart. PACT: Patient-Aligned Care Team.

This 3-year concurrent mixed-method implementation study employed a community-based participatory research perspective [7] using concurrent mixed-methods [12]. Table 1 provides an overview of implementation activities and deliverables/ outcomes. The study flow chart documents study progress over each of the three aims (Figure 1). We engaged PACT clinical partners in all aspects of this research. In Aim 1, we used qualitative semistructured focus groups and follow-up interviews to describe PACT members’ HIT experiences, needs, and suggestions for integrated use. In Aim 2, we conducted expert interviews and an environmental scan to identify existing implementation resources, develop, and formatively evaluate implementation strategies that reflect needs identified in Aim 1. In Aim 3, we proposed formative and summative methods to evaluate implementation outcomes. This study was approved by the University of South Florida Institutional Review Board. Participants did not receive incentives in alignment with VHA Office of Research Oversight regulation.

### Aim 1 Methods

We conducted focus groups and follow-up interviews with PACT members and extended PACT members. At each focus group, participants completed a questionnaire describing implementation strategies.

#### Sampling

We used a registry of all PACT members within Tampa VA (Florida) facilities, including one large hospital and 4 community-based outpatient clinics of varying size and geographic location, to develop a database. We compared the database with data from the VHA Support Service Center (VSSC) Transformation Initiative to identify individual PACT utilization level of SM. While our study targeted the full range of HIT resources, we used SM as a proxy indicator for HIT use because it is a valid direct measure for assessing HIT adoption among providers.

Our clinical partners indicated that monthly meetings are held with all PACTs. We held in-service presentations to engage PACT members during one of the meetings and introduced the study to all PACT members. An information sheet was provided to potential PACT participants during formal and informal meetings, posted in break rooms and other common areas, and shared through email. These information sheets provided an overview and purpose of the study and included study team contact information. This boosted PACT members’ awareness of the project; allowed introduction of clinical co-investigators, consultants, and champions; and promoted participant engagement as invested community members. We then sent individual email invitations to specific PACT members to participate in the study to include high- (top 25th percentile) and low-volume users (bottom 25th percentile) as measured by frequency of inbound and outbound SM and the ratio of patients assigned to SM with the PACT. Consultation with our clinical partners suggested some PACT members as high- or low-volume users who may not be readily identified by the secondary data sampling method (eg, patients may opt not to use SM). As such, we also overlapped the secondary data with their recommendations of perceived high- and low-volume HIT using PACT members to validate our sampling strategy and ensure recruitment of true high- and low-volume users.

The PACT members who were contacted were provided the choice of opting out of the study. Those who expressed interest were invited to participate in a focus group interview at their convenience. A subsample of those who participated was also contacted by telephone for an individual follow-up interview in person or by telephone. PACT members who were high- and low-volume HIT users expressed their desire to share their experiences and reasons for use/nonuse to create individual and organizational change to meet their personal and professional needs. We started with the core PACT members; however, during initial focus groups, we also solicited information about extended PACT members (eg, pharmacy, social work) and used snowball sampling to identify a subsample of extended PACT members.

#### Sample Size

Using purposive sampling, 65 PACT members (eg, physician, nurse, clinical associate, clerical associate, social work, pharmacy) in the Tampa VA hospital and community-based outpatient clinics, serving five counties, were recruited to participate in this study for a total of 21 focus groups. These focus groups included 10 high-volume, 9 low-volume, and 2 extended PACTs. In qualitative research, sample size relies on the quality and richness of information obtained [32,33]. Achieving conceptual saturation is the goal of qualitative research and is not dependent on sample size but rather the ability of the data to support interpretations [32,33]. Previous research by this team in this topical area reached thematic saturation between 20-30 interviews. We recruited to represent the facility type (hospital clinic vs community-based outpatient clinics). Based on emergent themes, we conducted 16 follow-up individual interviews with team members. We recruited the minimum sample necessary to represent the types of team member roles (ie, professional discipline) to compare experiences.

#### Measures

Participant data were collected using a participant demographic questionnaire and interview guides (see Table 2).

#### Data Collection Procedures

We used focus group interviews and follow-up individual interviews to complete data collection for Aim 1. Focus groups were used to collect data with PACT members (grouped by HIT volume status). Participants were scheduled to participate in groups at their facility for approximately 1 hour. Clinical partners recommended using the time that PACTs are given, one hour weekly, to do free-style staff activities. This promoted participation while not interfering in work and care delivery. Participants received an email before the focus group that included interview questions and some provider-focused HIT implementation content for review.

**Table 1:** Implementation plan activities and associated deliverables/outcomes.

Aim and activity			Deliverable/outcome
**Preimplementation**			
	**Aims 1-3**		
		Communicate the plan and begin process with PACT^a^ members	Investment across PACT/stakeholder groups Shared norms and expectations
		Include all representative groups in the planning process to get input	Investment across stakeholder groups Input from PACT/stakeholder groups
		Ensure strategies/goals are aligned with organizational & stakeholder goals	Investment across stakeholder groups Aligned strategies reflecting Diffusion of Innovations/PARiHS^b^ constructs
		Engage PACT leadership, consultants, and champions	Investment across stakeholder groups Setting expectation of organizational investment
	**Aim 1**		
		Conduct focus groups, follow-up interviews, and analysis	Data synthesis to inform implementation strategy development
		Set goal and strategy planning on timeline with PACT clinical partners	Implementation timeline aligned with expectations Matrix product that illustrates each audience, targeted strategies, with start date and duration
		Identify and train facilitators to identify champions to support strategy delivery	Points of contact designated Complete facilitator training for implementation
		Reiterate key measures and clear expectations	Planned outcomes data elements with stakeholders Align outcomes that reflect constructs and HIT^c^ use
		Plan visibility, integrated into regular activities	Integrate strategies with PACT activities (eg, staff meetings/professional development time)
		Address implementation program management needs with PACT	Action plan for activities, identify points of contact, deadlines, intermediate accomplishments, etc
	**Aim 2**		
		Conduct expert interviews and environmental scan	Collection of expert informant data and existing HIT implementation content and determination of need
		Implementation strategy development	Adaptation/development of strategies and content based on identified needs
		PACT member panel evaluation	Evaluation and revision of strategies and materials needed for implementation
**Implementation**			
	**Aims 1-3**		
		Prepare PACT members/stakeholders for implementation	Awareness of implementation activities Readiness across PACT/stakeholder groups
	**Aim 3**		
		Schedule implementation activities that align with PACT needs	Confirmed awareness of forthcoming implementation activities and readiness across PACT/stakeholders
		Conduct implementation activities that represent PACT needs	Primary pretest data collection to measure use of HIT and reflect implementation strategies
		Collect primary qualitative and secondary quantitative data	Implementation strategy feedback summary HIT use dataset
		Conduct follow-up with PACT members, other stakeholders (operational partners)	Primary posttest and secondary data collection Follow-up communication with PACT members, other stakeholders
			**Postimplementation**			
	**Aim 3**		
		Track implementation activities and outcomes and summarize progression of HIT use	Continue efforts on implementing initiatives Progress documented for continued efforts Continued use of community-based participatory research with PACT
		Recognize interim accomplishments and progress with PACT	Document and recognize accomplishments and milestones during implementation
		Conduct data analysis with invested PACT and stakeholders	Complete analysis of data with partnered input with PACT consultants and members
		Monitor & document lessons learned in efforts to increase HIT use with PACT members	Document lessons learned, approaches that work, those that need refinement, and adapt for future implementation in efforts
		Share study findings to PACT and stakeholder as recommended by feedback	Dissemination efforts supported by PACT member input to key audiences Reporting reflects pre-post measure changes

^a^PACT: Patient-Aligned Care Team.

^b^PARiHS: Promoting Action on Research Implementation in Health Services.

^c^HIT: health information technology.

**Table 2:** Aim 1 participant self-reported measures and characteristics.

Concept and measure			Psychometric properties
**Demographics**			
	Participant survey	16 self-report items to assess facility & unit; PACT^a^ role; length of time at facility, unit, in VHA^b^, in health care; age; gender; race; ethnicity; professional degree; licenses; computer/internet use; My Health*e*Vet status	
**Virtual medical modality use**			
	Participant survey	18 self-report items for each HIT^c^ including use count; HIT use; patient HIT use count, and relative advantage, compatibility, complexity, observability, context, facilitation constructs	
	Focus group script	Items to elicit information about concepts including clinical experience/evidence, personal and team factors and best practices; context, team, organizational, and environmental factors; and external/internal facilitation factors such as readiness for use, audit, feedback & reinforcement, leadership; advantages/ usefulness, compatibility, complexity/ease of use, observability of HIT. Items addressed PACT member perceptions of patients’ preferred communication methods, attempts to engage patients, and alternative resources for using HIT	
	Follow-up interview script	Items were driven by data collected in PACT focus groups to follow up on emergent themes and preferred dissemination methods	

^a^PACT: Patient-Aligned Care Team.

^b^VHA: Veterans Health Administration.

^c^HIT: health information technology.

Consent and data collection were conducted in a designated room at Tampa facilities to ensure confidentiality. Before beginning the focus group, each participant was asked to complete the demographic survey, and the focus group facilitator asked and received permission to audio record the discussion using VHA-approved equipment. The facilitator used the interview script to ensure that all topics were covered. Standard communication techniques were used to stimulate discussion, such as prompts, summarizing statements, silence, and eye contact. The facilitator also took written observational notes.

After completion of the focus groups, follow-up individual interviews were conducted with a purposeful subsample by phone or in person at the PACT member’s convenience. These interviews served multiple purposes. Though we addressed group dynamics in the focus groups, we needed to account for power differentials. We used individual interviews to ensure team members had an opportunity to communicate personal needs and experiences, discuss issues that may not have been fully addressed in the focus groups, explore themes that emerged in focus groups, discuss relevant issues based on the respondent’s PACT role, and explore the dynamic of using HIT within the context of the team. Methods like those used in focus groups were used to solicit information. Follow-up interviews allowed PACT members to review data cases and focus group summaries. The participants verified findings and provided additional input and/or clarification to ensure validity of Aim 1 findings from the PACT member perspective.

#### Data Analysis

Qualitative data collection and analysis progressed concurrently. In this way, insights from data analysis were used to iteratively guide subsequent data collection. The unit of analysis of the case study focused on the lived experiences of PACT members using HIT. These “cases” were analyzed using content analysis to identify patterns of similarities and differences that resulted in descriptions of relationships and recurring patterns of experience, behavior, and beliefs so that the phenomena could be understood within its cultural context. Analysis methods were used to identify domains and taxonomies related to provider data for focus groups and follow-up interviews [32]. This method involved inductive data reduction to distill the essential domains of participant experiences. Participant comments for case studies were organized to develop codes and merged to develop categories. Categories were compared and relationships were identified. Categories were grouped into a taxonomic structure. As coding schemas were developed to create domains and taxonomies, data samples were extracted and coded by two research team members and evaluated for interrater reliability and credibility. The datasets collected for each PACT member type were compared to one another to determine commonalities and differences. The research team conducted a complex matrix analysis to analyze across-group domains and taxonomies [34]. Descriptive and comparative matrices, which identify the patterns of regularities and inconsistencies were constructed. Comparative matrices provided an opportunity to identify the most relevant and representative components by HIT users. Representative cases were extracted from the dataset and analyzed for domains and themes, which were used to support strategy development. Focus group activities were entered into a Research Electronic Data Capture database and exported into a Microsoft Excel file. Response frequencies were analyzed statistically to determine which implementation strategies PACT members considered most and least useful for increasing uptake of technology. Once cases and comparative matrices were developed, follow-up interviews allowed PACT members and consultants to review these data to verify findings and provide additional input and/or clarification. Quantitative data including demographic survey data were summarized with descriptive statistics to describe sample characteristics.

### Aim 2 Methods

After conducting Aim 1, we developed PACT-focused implementation strategies using expert informant interviews, environmental scan, strategy development and evaluation, and the development of an implementation plan to address the PACT member needs identified in Aim 1. Our team, and identified champions, facilitated forums to (1) deliver core HIT information, (2) facilitate open group discussion of HIT specific implementation tools and best practices, and (3) identify champions and team members for follow-up contact and support. After the first forum, forums focused on trouble shooting barriers and discussing work-arounds relevant to HIT. Research team members met between forums with clinical investigators and champions to prepare and anticipate potential barriers and trouble shooting. Feedback received during these forums were documented as supplemental qualitative data memos in the qualitative Aim 3 dataset. We also developed a toolkit that was reviewed and distributed to all PACTs using multimedia. Other activities (eg, emails, presentations) were used.

#### Sampling

A subsample of participants from Aim 1 were invited to participate as panelists for a PACT Member Panel Evaluation. We approximated a subsample of minimum of 10 and a maximum of 15 PACT members to participate in this panel. These participants were selected based on study team members’ assessment of their enthusiasm to participate and their thoughtful responses during their focus group. Due to our observation of the limited ability of PACT members to visualize and articulate a vision of integrated proactive use of HIT across systems, the study protocol was amended to add a series of expert interviews with 13 clinical, operational, and administrative expert informants to assist the team in collecting more robust data that would contribute to the vision of integrated proactive use of HIT to deliver care within the VA.

#### Expert Informant Interviews

Expert informant interviews (n=13) were conducted by phone or in person. Aim 2 participants were recruited via email using the expert informant invitation email. In addition, a snowball technique was used in which the study team provided current participants with our PACT HIT study recruitment materials and contact information that they could pass on to other expert individuals who were also interested in participating. Expert informant interviews were conducted to answer the Aim 2 research questions. All interviews were recorded with permission. The interviewer used methods like those described in the focus group data collection procedures described above. Audio recordings were transcribed and analyzed using content analysis.

#### Environmental Scan

An environmental scan was conducted to answer Aim 2 research questions. Our team had ongoing partnerships with several electronic health (eHealth) researchers within the VA and had worked with several of these stakeholders during previous projects. We leveraged these existing relationships to complete Aim 2. We contacted these researchers and stakeholders to identify existing materials, as reviewed previously. To complete this aim, we distributed an initial email and made follow-up calls as needed to identify any new or emerging provider focused implementation strategies/tools to support HIT adoption. Though, some materials already existed for individual HIT, we identified the need to either adapt these or develop new materials and strategies to specifically address integrated use.

#### Patient-Aligned Care Team–Focused Implementation Strategies Development and Evaluation

We collaborated with operational, research, and PACT partners to collect resources to support PACT-focused HIT implementation strategy development and PACT member evaluation. We conducted a gap analysis based on Aim 1 findings and reviewed collected resources to determine what implementation strategies needed to be created or further developed. We drew from the bank of evidence-based implementation strategies [35], which supported adoption of an innovation that aligned with participant reports from Aim 1. Once strategy content and an implementation plan were developed, a PACT member panel provided a formative evaluation of content and finalized the implementation plan.

PACT Member Panel Evaluation was conducted with invested PACT consultants and team members who expressed willingness to participate on the panel to provide feedback on the implementation content and plan. As part of completing Aim 1, we identified key informants and invited them to participate on a panel to evaluate the implementation content. Once all materials were developed, panel members electronically received access to the content and evaluation form(s) to conduct a formal review of implementation content. To avoid reviewer burden, the number of products reviewed by each reviewer was minimized. Each panel member used the Implementation Content Evaluation Measure to evaluate their assigned implementation content. This measure consisted of three sections to facilitate evaluation: (1) relevance to virtual medical modalities, (2) relevance to implementation constructs, and (3) evaluation of content design and format. Scoring ranged from not applicable to strongly disagree. Once completed, evaluation forms were collected and collated, and a panel reviewer call/in-person meeting was scheduled to allow panel member discussion, collective review, and final synthesis of feedback and recommended revisions. Panel meeting type was determined by the member preference. Panel member participation took 3-5 hours total time.

#### Implementation Strategy Development

Implementation strategy development was conducted on receipt of all content. We conducted a gap analysis based on the needs identified in Aim 1. We developed a content review matrix to evaluate the materials to facilitate the gap analysis. The content review matrix addressed three primary sections of interest: (1) the degree to which the content addressed each HIT and their integrated use, (2) the degree to which the content addressed issues identified in Aim 1 relevant to implementation constructs including external environment, health care organization, site factors (ie, context) facilitation (eg, role clarity, supportive activities), patient factors, individual factors (eg, high- vs low-volume users, relative advantage, compatibility, complexity, observability), and (3) evaluation of the content design and format. Each evaluation item solicited feedback on how content could be improved to address evaluation elements. This tool was also used by the expert panel reviewers.

Guided by PACT clinical partner input, we adapted and developed content in collaboration with operational partners. We synthesized evidence-based strategies to create a more holistic approach to support HIT use as an integrated system. We employed a comprehensive set of strategies that were identified as evidence-based approaches to implementing change using core constructs from Diffusion of Innovation and PARiHS. We employed strategies including (1) audit and feedback (eg, HIT data elements were collected for PACTs to monitor, evaluate, and provide feedback), (2) dynamic virtual training/support (eg, HIT templates, virtual PACT telehealth materials, standalone videos, experiential learning test accounts), (3) educational meetings (eg, training/in-services at regularly scheduled bimonthly staff development meetings), (4) incentive structures (eg, increasing awareness about workload credit), and (5) tailored strategies (eg, PACT-driven development and evaluation of strategies and content as recommended by Powell et al [35]. We also employed emerging innovative strategies. As appropriate, these approaches were synthesized within a peer-to-peer story-telling context. We collaborated with PACT members to identify best practices focusing on how high-volume users leverage HIT within their workflow. Data have been previously collected related to use of SM in an ongoing collaborative operational project. We used this existing project as a road map for collecting best practices from high-volume users. We included these best practices in “forum” in-service trainings, story-telling videos, and other strategy content.

### Aim 3 Methods

After developing PACT-focused products to promote HIT adoption (intervention), we will conduct an evaluation using formative and summative methods in a pre-post single group design.

#### Sampling

All PACT members will be exposed to the intervention as part of monthly training and staff development meetings. We will conduct open recruitment with all 252 PACT members from the participating Tampa facilities (ie, hospital, 4 community-based outpatient clinics). Participants in Aim 2 will not participate in data collection for Aim 3.

#### Sample Size/Power Analysis

To answer the Aim 3 hypothesis, of the 252 PACT members, we anticipate a minimum participation rate of 50% in the pretest and posttest surveys (n=126). Because it is difficult to get clinicians with competing demands to complete questionnaires, we have calculated our expected sample size based on a worst-case scenario of a 25% response rate (n=63). We tested change scores on 8 separate subscales on the pretest/posttest measure. To control for hypothesis-wide error, we employed the Bonferroni adjustment and divided the nominal error rate of 5% by 8. A review of the literature did not identify a prior study to provide information about effect size, therefore we used the convention suggested by Cohen to compute the effect size [36]. Since a single group for each change score was measured before and after intervention, the effect size (Cohen *d*=0.50, medium effect size) was the mean difference between the pretest and posttest scores divided by the sample standard deviation of the change score [37]. A sample size of 63 achieves an 86% power to detect a difference in pre- and postintervention using a one-sample paired *t* test. We will use the rate of response for each strategy presented in Table 3 of each PACT member as outcome. We will have six timepoints preintervention and six timepoints postintervention for each implementation strategy. We expect separate slopes before and after the intervention. We will employ the simulation methods, to fit a piece-wise random effect model that includes separate pre- and postintervention slopes with an assumed effect size of 0.5 standard deviations from the mean [38,39]. A sample size of 63 (with 12 repeated values) with a type 1 error rate of 0.01, after Bonferroni adjustment, will give greater than 80% power for the analysis.

#### Measures

Participant data will be collected using a participant demographic survey and interview guides. Measure characteristics are presented in Table 3.

#### Data Collection Procedures

To answer Aim 3 research questions, participants will be asked to complete the questionnaire used in Aim 1 before and after the intervention followed by a brief debriefing interview. For Aim 3, secondary administrative data of HIT use from all PACTs in the Tampa hospital system 6 months pre- and postintervention will be obtained.

Links to electronic self-administered surveys via research electronic data capture will be emailed to each participant pre- (on consent) and postexposure (6 months post consent) to the implementation strategies as self-reported measures. Paper-pencil surveys will be provided as an option.

Debriefing interviews will be conducted in a group setting or as individual interviews, based on PACT member need. If the respondent is unable to stay, they will be given the option to conduct the debriefing by phone at another time of their convenience. All interviews will be recorded with permission.

**Table 3:** Aim 3 self-reported measure characteristics.

Concept and measure		Characteristics
**Demographic**		
	Participant survey	Described in Table 2
**Strategy effectiveness**		
	Participant survey	Described in Table 2
	Interview script	To elicit perceptions about strategies and materials, respondents were prompted to provide recommendations for improvement, and additional materials, formats, etc

The interviewer will use methods like those described in the focus group data collection procedure described above. The brief interview script will solicit respondents’ perceptions about the implementation strategies and materials and as well as recommendations on ways to improve and increase engagement, for example, “Did you think the intervention/strategy was useful to you for providing care to patients/daily workflow?” and “Did your participation/exposure impact your intention to use virtual care tools?”

We will collect administrative data to examine HIT use from local (ie, VetLink) and national administrative data sources (ie, VSSC, Corporate Data Warehouse [CDW]). Data will be collected for all PACTs in the Tampa VA hospital system 6 months pre- and postintervention. Secondary data collection will allow examination of changes in rates of HIT use (Table 4). We have identified multiple data sources to ensure we have options if data availability changes. We will also collaborate with operational partners to collect data elements from CDW. When data are received at the patient level, crosswalks will be used to connect patient level data to PACTs for team level analysis. If any data elements are housed for only 30-day increments, such as the MHV prescription refill data, we will collect data as often as needed, such as 30-day increments. Additionally, the study team has access to local VetLink Kiosk administrative data.

#### Data Analysis

Qualitative data analysis methods described in Aim 1 will be used to analyze debriefing interviews [32]. Quantitative data will be analyzed using the individual PACT member as the unit of analysis. We will use a one sample paired *t* test to compare pre- versus postintervention. To determine the association between the change scores across MHV implementation strategies, we will use Pearson correlation coefficient. If the response rate is higher than our conservative estimate, we will compare results for low- and high-use groups. We will use administrative data to calculate the rate of response for each implementation strategy of each PACT member (Table 4). For example, the number of patients per time interval within a PACT that opt to use SM divided by the number of PACT patients in the teams’ panel will represent the outcome. We will first conduct an exploratory analysis to evaluate the rate of responses separately for the pre- and postintervention and calculate summary statistics for differences in PACT pre- and post-averages.

**Table 4:** Aim 3 secondary data elements and data collection plan (data collected 6 months pre- and postexposure to implementation intervention).

Construct and variables			Measure		Data source
**Secure messaging use**					
	Registration	Number of patients registered		VSSC^a^ Compass PACT^b^ data cubes or VSSC Transformation Initiative data cube or Veteran and Consumer Health Informatics Office CDW^c^ data request	
	Authentication	Number of patients authenticated			
	Opted-in	Number of patients opted in			
	Inbound SM^d^	Number of inbound messages			
	Outbound SM	Number of outbound messages			
**MHV^e^ Rx refills use**					
	Prescription refill orders	Number of prescription refill orders		VSSC Transformation Initiative data cube or Veteran and Consumer Health Informatics Office data request	
**Telephone use**					
	Encounters	Number of encounters		VSSC Compass PACT data cubes	
**Home telehealth use**					
	Encounters	Number of encounters		CDW Telehealth Visits Report, including secondary codes for Home Telehealth, Clinical Video, Store & Forward	
	Visits	Number of visits			
	Unique patients	Number of unique patients			
**VetLink kiosk use**					
	Check-in	Number of patients checked in		Local VetLink Kiosk administrative data report	
	Demographic update	Number of patients who updated demographic data			
	Assistance required	Number of patients requiring help at kiosk			

^a^VSSC: VHA Support Service Center

^b^PACT: Patient-Aligned Care Team.

^c^CDW: Corporate Data Warehouse.

^d^SM: secure messaging.

^e^MHV: My Health*e*Vet.

We will use standard statistical tests to check for the departures of normality pre- and postintervention. As we expect to see separate slopes before and after the intervention, we will define a model that accommodates the two slopes and their difference using a piece-wise random effect model and separate pre- and postintervention slope in one model for each implementation strategy [40]. The results of the fitted model will consist of two main parts: a set of individual intercepts and two slopes (each representing pre- and postintervention). We will compare the pre- and postintervention slopes and if the difference is significantly different from zero, we will conclude that the pre- and postimplementation strategy was different. We will investigate effects of baseline covariates (eg, community-based outpatient clinics vs noncommunity-based outpatient clinics) that may influence changes in response rate over time.

## Results

Study enrollment for Aim 1 has been completed. Aims 1 and 2 data collection and analysis are underway. Aim 3 activities are scheduled for year 3.

## Discussion

### Principal Considerations

The goal of this study protocol was to identify effective implementation strategies for increasing PACT member awareness and motivation to use HIT proactively and in an integrated approach to better serve their patients and increase workflow efficiency. This protocol illustrates a community-based (ie, PACT) participatory approach to support clinical team members’ adoption and sustained integrated proactive use of HIT. To our knowledge, this protocol is unique in that it informs PACT-focused implementation strategies to support HIT use within a large health care system.

The proactive integrated use of HIT is a direct pathway to optimizing health care delivery to support patients’ ongoing health care needs. Participatory methods are a human-centered approach to engage clinical team members to identify focused implementation strategies that support their proactive integrated use of HIT to support health care delivery. The use of mixed-methods with critical stakeholders supports the development of targeted implementation approaches that will drive adoption and sustained use of HIT. From the end user perspective, proactive integrated use of HIT will increase individual proficiency and efficiency and ultimately optimize HIT value.

### Strengths and Limitations

This protocol contributes to the field in three distinct ways: (1) use of a community-based participatory approach, with primary care teams as the unit of analysis, (2) identification of primary care–focused implementation strategies to increase uptake and proactive integrated use of electronic health resources, and (3) use of secondary data to assess utilization of an enterprise-wide suite of electronic health resources. Primary care teams such as VA PACTs and community Patient-Centered Medical Homes are relatively new to US health care systems. Using them as a unit of analysis highlights the influence of team philosophy on behavior. Implementation strategies are varied. Focusing on primary care strategies tailors our findings to this unique segment of health care. Secondary data analysis of individual electronic health resource utilization uses a new data source to inform findings of this research as well as future eHealth-related research efforts.

This protocol also had limitations. First, the sample size was comparable to other qualitative studies [41], based on a representative sample of participants but may not be generalizable to other clinical groups. We purposively recruited PACT members as a base of community members representing primary care, as they can provide salient in-depth feedback; however, we may have missed valuable data that may represent other clinical groups. To address this limitation in part, we expanded recruitment to extended PACT members to include pharmacy, nutrition, mental health, and social work providers. Furthermore, the third aim was designed to develop a blueprint for secondary data collection to measure HIT use pre- and poststrategy implementation. Although the sample size was powered to determine intervention effects, findings will not be conclusive, as such a subsequent larger multisite study is warranted, with a more rigorous implementation study design, such as a step wedge design.

Second, this protocol was designed to develop implementation strategies designed to promote proactive integrated use of HIT. Because this is not common practice in delivering clinical care, PACT members recruited in Aim 1 had limited ability to visualize and articulate a vision of integrated proactive use of HIT across systems. As such, the study protocol was amended to add a series of expert interviews with clinical, operational, and administrative expert informants to assist the team in collecting data that would contribute to the vision of integrated proactive use of HIT to deliver care within the VA.

Future research should inform the continued support of clinical team member’s proactive integrated use of HIT resources, including both clinical team member user experiences and outcomes, representing diverse clinical groups. Clinical and organizational processes, including workflow should be explored and clearly identified. These continued efforts can guide the development and dissemination of best practices. In alignment with VA goals and the mission of VA’s Office of Connected Health, these data will support the adoption and sustained use of HIT to support clinical team delivery of personalized, proactive, and patient-driven health care [42].

### Conclusions

This protocol employed community-based participatory mixed-methods and multiple data sources to identify effective implementation strategies for increasing PACT member awareness and motivation to use HIT in a proactive integrated approach with patients. This protocol highlights the practical, technological, and participatory factors involved in facilitating implementation research designed to engage PACT clinical members in the proactive integrated use of HIT. Using this protocol, best practices can be identified and disseminated, leveraging PACT-focused strategies that reflect team member preferences to support subsequent implementation efforts at regional and national levels.

## Abbreviations

CDW: Corporate Data Warehouse
eHealth: electronic health
HIT: health information technology
MHV: My Health*e*Vet
PACT: Patient-Aligned Care Team
PARiHS: Promoting Action on Research Implementation in Health Services
RN: registered nurse
SM: secure messaging
VA: Department of Veterans Affairs
VHA: Veterans Health Administration
VSSC: VHA Support Service Center
